# MAM-mediated mitophagy and endoplasmic reticulum stress: the hidden regulators of ischemic stroke

**DOI:** 10.3389/fncel.2024.1470144

**Published:** 2024-11-21

**Authors:** Ziyi Jia, Hongtao Li, Ke Xu, Ruobing Li, Siyu Yang, Long Chen, Qianwen Zhang, Shulin Li, Xiaowei Sun

**Affiliations:** ^1^The First Clinical Medical College, Heilongjiang University of Chinese Medicine, Harbin, China; ^2^The First Affiliated Hospital, Heilongjiang University of Chinese Medicine, Harbin, China; ^3^The Second Clinical Medical College, Heilongjiang University of Chinese Medicine, Harbin, China

**Keywords:** mitochondrial-associated endoplasmic reticulum membrane (MAM), mitophagy, endoplasmic reticulum stress, ischemic stroke (IS), unfolded protein response (UPR)

## Abstract

Ischemic stroke (IS) is the predominant subtype of stroke and a leading contributor to global mortality. The mitochondrial-associated endoplasmic reticulum membrane (MAM) is a specialized region that facilitates communication between the endoplasmic reticulum and mitochondria, and has been extensively investigated in the context of neurodegenerative diseases. Nevertheless, its precise involvement in IS remains elusive. This literature review elucidates the intricate involvement of MAM in mitophagy and endoplasmic reticulum stress during IS. PINK1, FUNDC1, Beclin1, and Mfn2 are highly concentrated in the MAM and play a crucial role in regulating mitochondrial autophagy. GRP78, IRE1, PERK, and Sig-1R participate in the unfolded protein response (UPR) within the MAM, regulating endoplasmic reticulum stress during IS. Hence, the diverse molecules on MAM operate independently and interact with each other, collectively contributing to the pathogenesis of IS as the covert orchestrator.

## 1 Introduction

Stroke ranks as the second most common reason for disability and mortality worldwide (Saini et al., 2021), with Ischemic stroke (IS) responsible for the majority of new cases, accounting for around 87% of all instances (Kleindorfer et al., 2021). IS occurs when there is a decrease in blood flow, resulting in a shortage of oxygen and glucose in the brain tissue. This triggers a series of responses (Shi et al., 2021), including endoplasmic reticulum (ER) stress, mitochondrial autophagy, and cell ion overload, all of which can contribute to neuronal death, worsen the progression of cerebral ischemic injury, and impact the final outcome (Noh et al., 2023). At present, the primary focus of treating IS is on restoring blood flow, especially by using thrombolytic therapy within a 3–4.5 h window to reverse reversible ischemic damage. However, due to the limited treatment timeframe and the resulting reperfusion injury, patients with IS often experience unfavorable outcomes (Liu et al., 2023). After the passing of this time frame, it becomes impossible to repair damaged neurons, and reperfusion could result in cerebral ischemia/reperfusion injury. This is mainly caused by ER stress and mitochondrial autophagy (Dhapola et al., 2024), which further worsens cell death and brain damage. Hence, it is crucial to investigate effective therapeutic approaches that can prolong the treatment window and enhance results for individuals suffering from IS.

The mitochondria-associated endoplasmic reticulum (MAM) acts as a channel that facilitates communication between the ER and mitochondria. It consists of substructures within the ER, the outer mitochondrial membrane (OMM), and a set of proteins that establish a dynamic connection (Yang et al., 2020). Hung et al. identified 634 proteins on the ER and 137 proteins on mitochondria. They found that 68 of these proteins overlapped with MAM when comparing them (Hung et al., 2017). The initial estimation of the distance between the ER and the outer membrane of the mitochondria was approximately 100 nanometers. Nevertheless, a growing body of research indicates that the configuration of MAM is not fixed; instead, it is fluid and capable of adapting to various cellular physiological and pathological circumstances. The distance between the rough ER and the outer membrane of the mitochondria typically ranges from 10 to 100 nm (Giacomello and Pellegrini, 2016), with a smooth ER gap width of 10–15 nm and a rough ER gap width of 20–30 nm (Csordás et al., 2006). Artificially disrupting this contact can result in ER stress (Bravo et al., 2011).

**Figure 1:**
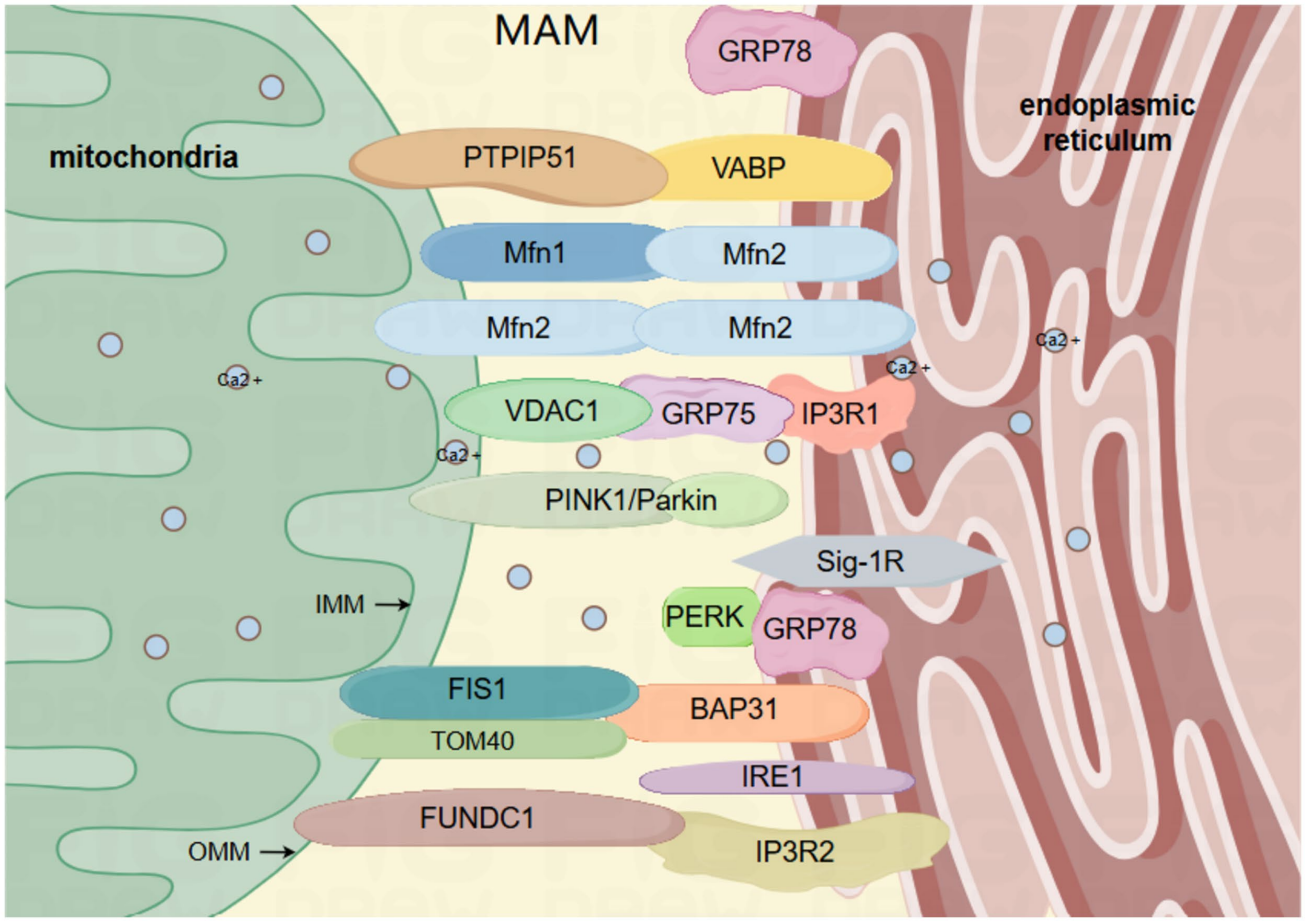
The primary framework of MAM: the mitochondrial associated membrane (MAM) comprises the outer and inner membranes of the mitochondria, and is abundant in diverse proteins that are interconnected by tethering proteins, such as VAPB-PTPIP51, IP3R-GRP75-VDAC1, BAP31-FIS1, FUNDC1-IP3R2, MFN2-MFN1/2. VAPB, vesicle-associated membrane protein-related protein B; PTPIP51, Protein Tyrosine Phosphatase Interacting Protein 51; IP3R, inositol 1,4,5-trisphosphate receptor; GRP75, glucose-regulated protein 75; GRP78, glucose-regulated protein 78; VDAC1, voltage-dependent anion channel 1; BAP31, B cell receptor-associated protein 31; FIS1, mitochondrial fission protein 1; FUNDC1, protein 1 with FUN14 domain; MFN1/2, mitofusin 1/2; IRE1, inositol 1,4,5-trisphosphate 3-kinase 1; Sig-1R, sigma-1 receptor; PINK1, serine/threonine kinase; Parkin, E3 ubiquitin ligase; mitochondrial outer membrane translocase component TOM40; PERK, protein kinase R-like ER kinase. By Figdraw.

Undoubtedly, it has been established that MAM serves as a focal point for transmitting stress signals from the ER to the mitochondria, particularly when ER protein homeostasis is compromised (van Vliet and Agostinis, 2018). Due to its role as a signal transduction platform, MAM primarily depends on proteins to carry out its diverse functions. As a result, numerous studies categorize the fundamental elements of MAM based on their primary roles. For instance, the transportation of calcium involves glucose-regulated protein 75 (GRP75), mitochondrial voltage-dependent anion channel (VDAC1), and inositol 1,4,5-trisphosphate receptor (IP3R) (Tubbs et al., 2014). Cell apoptosis involves BAP31, a protein linked to the B cell receptor, and FIS1, a homolog of mitochondrial fission 1 (FIS1) (Saito and Imaizumi, 2018). ER stress involves the sigma-1 receptor (Sig-1R), inositol-requiring enzyme 1 (IRE1), protein kinase R-like ER kinase (PERK) (Saito and Imaizumi, 2018), and glucose-regulated protein 78 (GRP78) (Eysert et al., 2020). Autophagy involves VAPB (vesicle-associated membrane protein B), PTPIP51 (protein tyrosine phosphatase interacting protein 51), MFN1/2 (mitofusin 1/2), TOM40 (translocase of OMM component), PINK1 (serine/threonine kinase), Parkin (E3 ubiquitin ligase), and FUNDC1 (protein with FUN14 domain) (Ji et al., 2022; Lv et al., 2022). The mitochondria and ER play crucial roles as cell organelles in eukaryotic cells. In mammalian cells, the surface of mitochondria is approximately 5–20% parallel to the ER (Rizzuto et al., 1998). This intimate structural connection enables the ER to respond to diverse stress stimuli and transmit stress signals to mitochondria (Iurlaro and Muñoz-Pinedo, 2016). In a similar manner, mitochondria have the ability to send signals to the ER, ensuring that compensatory responses or cell death events are effectively carried out. This system of signal transduction is typically examined as a relatively independent subcellular organelle structure referred to as the MAM (Figure 1). In conclusion, MAM plays a crucial role in maintaining cellular homeostasis and biological functions by acting as an imperceptible regulator that governs mitochondrial autophagy and ER stress.

## 2 MAM modulates mitophagy to mitigate IS

Mitochondrial autophagy is a specific type of autophagy capable of removing impaired or ineffective mitochondria. Mitochondrial autophagy is activated in the aftermath of an IS. Research has indicated that in the context of IS, mitochondrial dysfunction can result in an increased release of pro-apoptotic factors, ultimately leading to cell death within the affected area (Jürgensmeier et al., 1998). In the reperfusion phase, mitochondrial autophagy mainly plays a protective role (Kumar et al., 2016). Hence, there is a widespread belief that moderate mitochondrial autophagy provides a protective effect, whereas an excessive level of mitochondrial autophagy could potentially worsen neuronal demise (Mo Y. et al., 2020).

MAM plays a crucial role in initiating and carrying out autophagy, and it contributes to the formation of autophagosomes. Interestingly, MAM can play both positive and negative roles in autophagy under various physiological or pathological conditions, a phenomenon closely associated with the intricate composition of its constituent proteins and their multifaceted functions (Ji et al., 2022). The PINK1/Parkin pathway is widely recognized as a crucial regulator of mitochondrial autophagy. Recent studies have confirmed the accumulation of PINK1 and Parkin in MAM (Van Laar et al., 2015). In the process of mitochondrial autophagy, PINK1 and Beclin1 are observed to gather on the surface of MAM, facilitating the establishment of ER-mitochondria connections and the formation of autophagosomes (Gelmetti et al., 2017). FUNDC1 has the ability to function as a receptor for mitochondrial autophagy during hypoxia, regulating both mitochondrial fission and mitochondrial autophagy at MAMs. This ultimately strengthens its association with mitochondrial processes (Wu et al., 2016b). Moreover, prior research has demonstrated that Mfn2 depletion disrupts MAM and results in compromised autophagosome formation (Gomez-Suaga et al., 2017). All of these observations indicate an indisputable connection between MAM and mitophagy. Hence, considering the diverse roles of mitophagy at various stages of IS, it is imperative to target MAM at the appropriate time for intervention in stroke regulation (Table 1).

**Table 1:** The role of PINK1/Parkin in IS.

Intervention strategies	PINK1/Parkin	Mitochondria autophagy	IS	References
Hydrogen	PINK1↑, Parkin↑	Activate	It offers neuroprotective effects for injured nerves	Wu et al. (2018)
Electroacupuncture	PINK1↑, Parkin↑	Activate	Reducing Neuronal Damage in Cerebral Ischemia–Reperfusion	Wang et al. (2019)
Garciesculenxanthone B	PINK1↑, Parkin↑	Activate	Preventing cerebral ischemia–reperfusion injury	Wu M. et al. (2021)
Rehmapicroside	PINK1↓, Parkin↓	Inhibition	Improving cerebral ischemia–reperfusion injury	Zhang Y. et al. (2020)
Neuropeptide	PINK1↑, Parkin↑	Activate	Participate in the neuroprotective effects of cerebral ischemia–reperfusion	Shao et al. (2021)
Peroxidase protein↓	PINK1↑, Parkin↑	Activate	It exacerbates cerebral ischemia–reperfusion injury and augments neuronal apoptosis	Hong et al. (2022)
Remote ischemic preconditioning	PINK1↑, Parkin↑	Activate	Preventing Cerebral Ischemic Injury	Jiao et al. (2022)
Neuroceramide analog protein↑	PINK1↑, Parkin↑	Activate	Reversing neurological damage caused by cerebral ischemia–reperfusion	Huang et al. (2022)
Docosahexaenoic acid	PINK1↑, Parkin↑	Activate	Protect neurons and reduce brain damage	Sun et al. (2022)

### 2.1 PINK1/Parkin in MAM and mitophagy

The PINK1/Parkin pathway is a highly intricate regulatory mechanism, and studies have shown that both PINK1 and Parkin are situated in the MAM under normal physiological conditions as well as during cellular stress (Barazzuol et al., 2020). Following an IS, detrimental stimuli such as ROS and hypoxia facilitate the initiation of PINK1 via phosphorylation. Phosphorylated PINK1 subsequently attaches to the surface of mitochondria, leading to the binding and phosphorylation of Parkin on the outer membrane of mitochondria. Upon activation, Parkin will partially attach ubiquitin to the OMM protein, thereby triggering mitophagy. Certain mitochondrial proteins undergo ubiquitination and subsequent degradation, a process crucial for mitophagy (Tanaka et al., 2010). Other ubiquitinated proteins are recruited by autophagy adaptor proteins, such as optineurin (OPTN) and nuclear dot protein 52 (NDP52), which anchor marked mitochondria to autophagosomes via their LC3-interacting region (LIR) motif, thereby initiating mitophagy (Lazarou et al., 2015). The main site where LC3-II is locally accumulated and the membrane origin of the mitochondria has been identified as MAM (Yang and Yang, 2013). Post-ischemic reperfusion therapy enhances mitochondrial autophagy through the induction of mitochondrial ubiquitination mediated by PINK1/Parkin, thereby mitigating transient global cerebral ischemia and ameliorating neuronal injury (Wen et al., 2021). Following mitochondrial depolarization, PINK1 and Parkin are recruited to the MAM, and it seems that the translocation of PINK1 to MAM is crucial for recruiting the autophagy machinery to this region (Gelmetti et al., 2017). When mitophagy is triggered, the amount of PINK1 rises in a manner dependent on calcium, and the expression of PINK1 is stimulated while mitophagy is initiated when there is a local increase in calcium levels during the exchange of calcium between cellular organelles in MAM (Gómez-Sánchez et al., 2014). Under typical conditions, there exists a delicate equilibrium between the generation and elimination of ROS within the human body. However, this balance is disrupted in the event of an IS. ROS serves as the primary initiator of mitochondrial autophagy, with excessive ROS triggering this process. The PINK1/Parkin pathway has the capacity to impede ROS accumulation and promptly eliminate damaged mitochondria, thereby regulating IS and substantially mitigating oxidative harm (Wen et al., 2023). IS-induced neuronal injury is partly due to the excitatory toxicity of glutamate (L-Glu), and it has been observed that the PINK1/Parkin pathway safeguards neurons against L-Glu-induced damage by maintaining mitochondrial function (Dou et al., 2022). It is important to mention that the recently identified extended form of PTEN (PTEN-L) has been demonstrated to function as a suppressor of mitophagy by obstructing Parkin’s movement to mitochondria and restraining its E3 ligase activity. Some PTEN has been identified to be localized on the ER membrane and in MAM (Wang et al., 2018a). Prior research has indicated that the activation of mitophagy occurs via the PINK1/Parkin pathway in instances of brain ischemia and reperfusion-induced brain injury (Lan et al., 2018). Therefore, the regulation of mitochondrial autophagy mediated by PINK1/Parkin in MAM may represent a promising approach for the treatment of IS.

### 2.2 FUNDC1 in MAM and mitophagy

FUNDC1 functions as a newly discovered MAM protein essential for triggering mitochondrial autophagy under hypoxic conditions. Research has indicated that FUNDC1 has the ability to interact with MAM-specific proteins, facilitate the generation of MAM, and influence mitochondrial dynamics (Wu et al., 2017). Under typical oxygen levels, a minimal quantity of FUNDC1 can be found within the MAM (Wu et al., 2016a). In the absence of oxygen, FUNDC1 accumulates at the MAM through its interaction with the ER resident protein CANX (calreticulin). The regulation of FUNDC1 mitochondrial autophagy is influenced by a variety of stress factors and cellular proteins. The phosphorylation of FUNDC1 at Tyr18 and Ser13 is carried out by Src and casein kinase 2 (CK2), respectively, which serves to inhibit its interaction with LC3 and the initiation of autophagy (Chen et al., 2016). In neurons undergoing ischemic reperfusion, excessive activation of Src due to ischemic injury leads to the phosphorylation of Tyr18 on FUNDC1. This results in the impairment of FUNDC1’s ability to bind with LC3, ultimately hindering its participation in neuronal mitochondrial autophagy. Repressing Src activity can preserve the function of FUNDC1 and initiate neuroprotective effects mediated by mitochondrial autophagy through FUNDC1 (Tang et al., 2023). FUNDC1 has the ability to engage with OPA1, a mitochondrial fusion protein, as well as Drp1, a mitochondrial fission protein (Chen et al., 2016). The breakdown of OPA1 at the S1 site caused by cerebral ischemia worsens the splitting of mitochondria and reperfusion damage in neurons (Li et al., 2022). Inhibition of Drp1 may restrict brain injury following IS and avert neuronal impairment and mortality (Grohm et al., 2012). FUNDC1 may enhance mitophagy and ameliorate neuronal damage following IS through its interaction with OPA1 and Drp1. Prior research has indicated a clear link between MAM and FUNDC1 in facilitating the process of mitochondrial autophagy, FUNDC1 interacts with another MAM protein, IP3R2, to mediate IP3R2-dependent calcium signaling from the ER to the mitochondria (Wu et al., 2017), thereby enhancing calcium transport in MAM and impacting mitophagy. An increasing number of studies are demonstrating the potential for regulating FUNDC1-mediated mitochondrial autophagy to improve IS (refer to Table 2). These findings indicate that targeting FUNDC1-mediated mitochondrial autophagy in MAM may hold promise for the treatment of IS.

**Table 2:** The role of FUNDC1 in IS.

Intervention strategies	FUNDC1	Mitochondria autophagy	IS	References
Tissue-type plasminogen activator	FUNDC1↑	Activate	Protect neurons from cerebral ischemia–reperfusion injury	Cai et al. (2021)
Electroacupuncture at GV20 and DU26	FUNDC1↓	Inhibition	Reduce cerebral ischemia–reperfusion injury and mitochondrial damage.	Tian et al. (2022)
Transcription factor forkhead box P1↑	FUNDC1↑	Activate	Reduce brain injury during cerebral ischemia–reperfusion period.	Li et al. (2023)
Unc-51-like autophagy activating kinase 1↑	FUNDC1↑	Activate	Prevent neuronal cell apoptosis induced by oxygen deprivation.	Wang et al. (2018b)

### 2.3 Beclin1 in MAM and mitophagy

While Beclin 1 was initially investigated for its role in regulating autophagy, it also contributes to the regulation of mitochondrial autophagy. After stimulating mitochondrial autophagy, it was translocated to the MAM, facilitating the crosstalk between the ER and mitochondria and boosting the efficacy of mitochondrial autophagy (Gelmetti et al., 2017). Beclin1’s localization on MAM ensures that the initiation of autophagosome formation occurs in close proximity to impaired mitochondria, facilitating their efficient engulfment. Beclin 1 collaborates with different cofactors to regulate the function of the Vps-34 protein and promote the formation of the Beclin 1-Vps34-Vps15 core complex, thus initiating autophagy (Kang et al., 2011). Furthermore, Beclin1 initiates mitophagy by promoting the translocation of Parkin from the cytosol to the mitochondria. It is noteworthy that Beclin1 is significantly upregulated by ischemic preconditioning (Xie et al., 2018), whereas its expression is downregulated following reperfusion injury (Dai et al., 2017). After cerebral ischemia, the primary localization of Ulk1 is in the small glial cells within the infarcted region, where it facilitates the development of anti-inflammatory pathways in these cells (Xiong et al., 2024). Beclin1 ensures the accurate targeting of autophagosomes to mitochondria via ULK1-dependent serine phosphorylation (Quiles et al., 2023), and Beclin1 may facilitate mitochondrial autophagy, thereby promoting neuronal repair following ischemic brain injury. Mouse studies have demonstrated that Beclin1 safeguards mitochondria by preferentially triggering a distinct mitophagy pathway. The protective role of Beclin1 in this process may potentially extend to MAM involvement, possibly contributing to the formation or maintenance of MAM (Sun et al., 2021). A recent investigation has presented fresh findings indicating that Beclin1 and Beclin2 exhibit around 57% sequence similarity and possess several common structural domains. However, it was observed that Beclin2 does not contain the Ulk1 phosphorylation site found in Beclin1 and is not localized to MAM during mitophagy (Quiles et al., 2023). These results provide additional evidence that the distinctive N-terminal domain of Beclin1 and its precise positioning in MAM could account for its distinct role in mitophagy. Research has demonstrated that the downregulation of Beclin1 can attenuate cell death in the ischemic hemisphere, facilitate neurogenesis, and diminish infarct size (Zheng et al., 2009). Repressing Beclin1 can impede the formation of autophagosomes, leading to a notable decrease in neuronal loss, glial proliferation, and cell death (Xing et al., 2012). In conclusion, Beclin1-mediated mitochondrial autophagy in MAM represents a promising therapeutic target for the management of IS.

### 2.4 Mfn2 in MAM and mitophagy

Mfn2 can be found in the MAM and serves as a prototypical functional tethering protein within this region. Its presence in the MAM allows for interaction with mitochondria, facilitating the formation of an organelle bridge within the cell (McFie et al., 2016). In the context of cerebral ischemia–reperfusion, the lack of Mfn2 hinders platelet activation, formation of prothrombotic platelets, and diminishes the extent of the infarct (Jacob et al., 2024). Mfn2 is essential for the proper functioning of mitochondria, encompassing fusion, axonal transport, inter-organelle communication, and mitophagy (Stuppia et al., 2015). Mfn2’s regulation of mitochondrial function can attenuate hypoxia-induced cell apoptosis (Peng et al., 2015). Downregulation of Mfn2 results in impaired mitochondrial function, disrupting calcium homeostasis and ultimately leading to delayed neuronal cell death. Studies have indicated a notable decline in Mfn2 levels 90 min after 6 h of middle cerebral artery occlusion. As a result, the decrease in Mfn2 is considered to occur later during reperfusion. Targeting this reduction may contribute to minimizing ischemic damage and widening the current limited stroke treatment window (Martorell-Riera et al., 2014). In order for mitophagy to take place, mitochondria need to undergo fission initially. This process involves the separation of dysfunctional mitochondria through mitochondrial fission and selective fusion, enabling their removal via mitophagy (Twig et al., 2008). Mfn2 is involved in the regulation of the structure of MAM and mitochondrial function, which is essential for protecting neurons. Decreased expression of Mfn2 results in impaired MAM function and mitochondrial activity, ultimately leading to increased fragmentation of mitochondria (Zhu et al., 2024). Ubiquitination of Mfn2 could potentially inhibit mitochondrial fusion by facilitating the breakdown of these proteins through proteasomes or by obstructing the formation of MFN dimers between mitochondria (Joaquim and Escobar-Henriques, 2020). Cerebral ischemia–reperfusion injury results from inadequate autophagy, and Mfn2 has the potential to enhance ischemia–reperfusion injury by boosting the generation of autophagosomes and facilitating their fusion with lysosomes (Peng et al., 2018). Additionally, the interaction between the ER and mitochondria occurs as an initial stage in the process of mitophagy, preceding the engulfment of cellular organelles by the autophagosome. Mfn2, a protein that tethers mitochondria to the ER, is essential for the formation of autophagosomes during mammalian starvation (Naon et al., 2016). Knockout or silencing of Mfn2 results in the separation of ER and mitochondria (de Brito and Scorrano, 2008). Studies have indicated a connection between global cerebral ischemia and the swift migration of Mfn2 from the mitochondria to the cytosol (Klacanova et al., 2019). Additionally, Mfn2 serves as a substrate for parkin, and during the initial phases of mitophagy, parkin may attach to ubiquitinated Mfn2 by interacting with the pUb domain. The ubiquitination of Mfn2’s HR1 domain by parkin is essential for efficient mitophagy (McLelland et al., 2018). Recent research has shown that Mfn2 is directly involved in inhibiting mitochondrial autophagy by connecting mitochondria to the ER (Basso et al., 2018). These studies indicate that targeting Mfn2-mediated mitochondrial autophagy in MAM may be an effective approach for regulating IS.

## 3 MAM modulates endoplasmic reticulum stress in IS

The ER plays a crucial role as a cellular organelle, overseeing the synthesis, transportation, and regulation of calcium levels within the cell. Following an IS, the regular function of the ER is disturbed, potentially leading to the onset of ER stress. During cerebral ischemia, the occurrence of ER stress is associated with local acute ischemia and neuronal damage in brain tissue. The stress in the ER leads to an adaptive reaction known as the unfolded protein response (UPR), which serves primarily as a survival-promoting mechanism aimed at reinstating ER homeostasis. Nevertheless, when cells undergo extended and persistent ER stress, the UPR directs them toward cellular apoptosis (Lenna et al., 2014). During the initial phase of UPR, MAM’s dynamic assembly takes place, a process typically viewed as supportive of cell survival. This is linked to heightened mitochondrial calcium absorption and improved respiration (Bravo et al., 2011). The MAM component has a strong connection with UPR, and essential elements of UPR like PERK and IRE1α have been identified within MAM. When the ER is under stress, there is an accumulation of IRE1α in MAM (Carreras-Sureda et al., 2017). Hence, targeting MAM-mediated ER stress could be a promising therapeutic strategy for IS (Tables 3, 4).

**Table 3:** The role of Beclin1 in IS.

Intervention strategies	Beclin1	Mitochondria autophagy	IS	References
miRNA-30a↓	Beclin 1↑	Activate	Reduce cerebral ischemic injury	Wang et al. (2014)
Chloride Channel-3↑	Beclin 1↑	Activate	Preventing cerebral ischemia–reperfusion injury	Zhang B. et al. (2020)
Melatonin	Beclin 1↓	Inhibition	Preventing cerebral ischemia–reperfusion injury	Lin et al. (2023)
Brain-derived neurotrophic factor infusion	Beclin 1↑	Activate	Ensure neuron survival and reduce the size of the infarcted area.	Yilmaz et al. (2024)
The 18-kDa translocator protein ↓	Beclin 1↓	Inhibition	Reduce neuronal cell damage	Mahemuti et al. (2023)
dichloromethane fraction of *Piper nigrum* L. and *P. longum* L.	Beclin 1↓	Inhibition	Reduce neuronal autophagy and exert neuroprotective effects.	Yuan et al. (2023)
Normal Pressure Oxygen Therapy	Beclin 1↓	Inhibition	Reduce the area of infarction	Wang et al. (2021)
Sphingosine kinase 2	Beclin 1↑	Activate	Protect neurons from ischemic injury	Song et al. (2017)

**Table 4:** The role of Mfn2 in IS.

Intervention strategies	Mfn2	Mitochondria autophagy	IS	References
Nuclear receptor subfamily 4 group A member1↓	Mfn2↑	Activate	Reinforce pro-survival signaling to mitochondria following brain IR injury.	Zhang and Yu (2018)
Electroacupuncture at GV20 and GV24	Mfn2↑	Activate	Reinforce mitochondrial fusion and diminish the extent of infarction.	Liang et al. (2023)
Cannabidiol	Mfn2↑	Activate	Reinforcing the protection of cerebral neurons against ischemic injury.	Xu et al. (2023)
Bioactive compound phelligridimer A	Mfn2↑	Activate	Recover mitochondrial function and mitigate cerebral ischemic injury.	Li et al. (2024)
Preprocessing for Sports Data	Mfn2↑	Activate	The infarcted area was reduced in size and there was an improvement in neurological deficits.	Qin et al. (2023)
USP30 ↑	Mfn2↑	Activate	Prevent mitochondrial fragmentation.	Chen et al. (2021)
small molecule echinacoside	Mfn2↑	Activate	Enhance mitochondrial fusion to enhance recovery from brain injury.	Zeng et al. (2021)

### 3.1 GRP78 in MAM and endoplasmic reticulum stress

**Figure 2:**
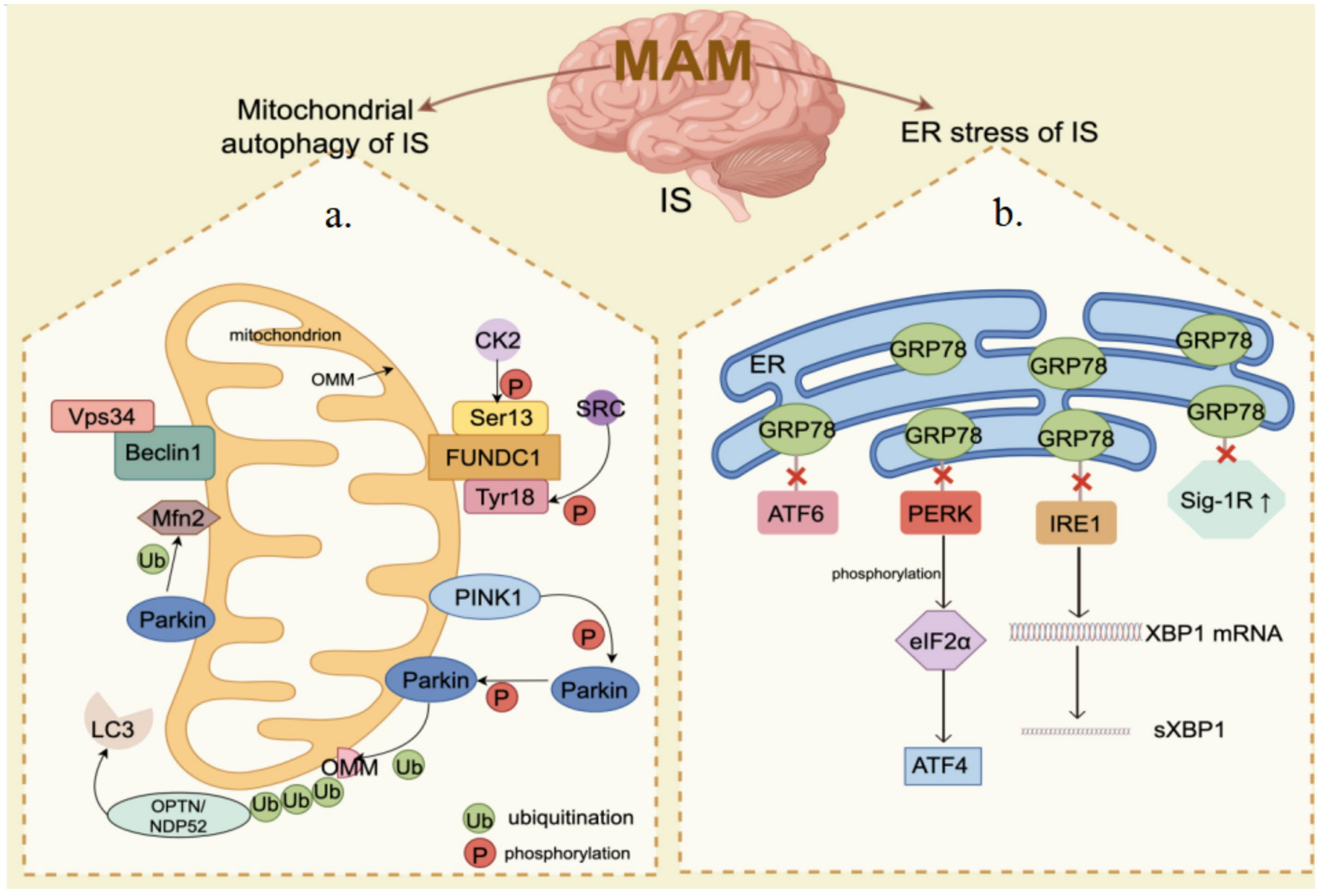
MAM mediates mitochondrial autophagy and endoplasmic reticulum (ER) stress in ischemia–reperfusion (IS). **(a)** Mitochondrial Autophagy: PINK1/Parkin, FUNDC1, Beclin1, and Mfn2 are significantly enriched in the MAM. When IS happens, the PINK1/Parkin pathway is activated, inducing mitochondrial autophagy through the ubiquitination of Mfn2 and other substrates. Beclin1 facilitates mitochondrial autophagy by interacting with Vps34. The phosphorylation status of FUNDC1 critically influences mitochondrial autophagy under hypoxic conditions. **(b)** ER Stress: The core components of the UPR, including IRE1, PERK, and ATF6, are also enriched in the MAM. Under ER stress conditions, dissociation of GRP78 from UPR leads to activation of PERK and IRE1 as well as upregulation of Sig-1R, which subsequently impacts ER function and cell survival. By Figdraw.

Glucose regulation protein 78 (GRP78) functions as an ER chaperone located within the intraluminal space and is a significant constituent of MAM (Prasad et al., 2017; Mahamed et al., 2023). The upregulation of GRP78 serves as an indicator of ER stress, regulating the activation of transmembrane ER stress sensors by means of binding and release mechanisms (Lee, 2005). GRP78 serves as the primary controller of the UPR, facilitating proper protein folding and inhibiting the accumulation of protein aggregates within the ER. UPR is controlled by three nearby detectors IRE1, PERK, and ATF6. GRP78 interacts with these three sensors via its peptide-binding domain, maintaining them in a state of inactivity. When cells accumulate misfolded proteins, they attach to GRP78 and interfere with its communication with the stress sensors located upstream. Moreover, GRP78 is a protein that is activated under stress and has broad expression in the cells of animals (Chang et al., 1987). When cells experience ER stress, particularly during the depletion of stored calcium and the buildup of irregular proteins, there is an increase in the transcription rate of GRP78 (Li et al., 1994). IS can result in the disruption of ER calcium homeostasis, damage to UPR, and impairment of proteasome function, thereby leading to secondary ER dysfunction. The sole method to break free from this potentially life-threatening pattern is to initiate UPR, thus prompting the production of GRP78 at a degree adequate for reconfiguring misshapen proteins (Paschen, 2004). The serine protease tPA is considered the standard treatment for cerebral ischemia, and prior research has identified Grp78 as a novel neural receptor for tPA. Activation of tPA by Grp78 on cell surfaces triggers a negative feedback loop that inhibits the activation of the PERK branch, leading to decreased phosphorylation of eIF2α and ultimately reducing neuronal death in cerebral ischemia (Louessard et al., 2017). Moreover, a research study indicates that GRP78 plays a role in facilitating the movement of peptides through the ER membrane and serves to safeguard cells against cell death induced by ER stress (Rao et al., 2002). An increasing number of research findings indicate the essential involvement of GRP78 in ER stress (refer to Table 5). In conclusion, GRP78 located in the mitochondrial associated membrane (MAM) is capable of regulating neuronal death induced by ER stress. Targeting GRP78 in MAM may represent a promising therapeutic approach for managing ER stress following IS (Figure 2).

**Table 5:** The role of GRP78 in IS.

Intervention strategies	GRP78	ER stress	IS	References
Ischemic preconditioning	GRP78↑	Diminish	Postponing the apoptosis of neurons	Hayashi et al. (2003)
Electroacupuncture at GV14 and GV20	GRP78↑	Diminish	There is a clear neuroprotective effect.	Chen et al. (2014)
long non-coding RNA KCNQ1OT1	GRP78↑	Diminish	The extent of brain tissue impacted by ischemia diminishes, leading to a reduction in cellular apoptosis.	Yao et al. (2014)
Gualou Guizhi Decoction	GRP78↑	Diminish	Reduce the rate of apoptosis in ischemic cortical neurons.	Chen et al. (2022)
Low temperature therapy	GRP78↑	Diminish	Enhance neuron survival	Aoki et al. (2001)
Simvastatin	GRP78↑	Diminish	Neuroprotective effect	Urban et al. (2009)
Cerebral ischemic post-treatment	GRP78↑	Diminish	Minimize cellular programmed cell death and safeguard the brain against injury caused by cerebral ischemia–reperfusion.	Yuan et al. (2011)
Remote ischemic postprocessing	GRP78↑	Diminish	Reduce cerebral ischemic injury	Liu et al. (2014)
Biochanin A	GRP78↑	Diminish	Repress cellular apoptosis and alleviate injury induced by cerebral ischemia–reperfusion.	Guo et al. (2021)
Electroacupuncture at GV20 and ST36	GRP78↑	Diminish	Protect cells from neuronal damage.	Zhang et al. (2021)

### 3.2 IRE1 in MAM and endoplasmic reticulum stress

The transmembrane protein IRE1, which is involved in the UPR, possesses both a kinase domain and an RNAse domain (RD) and is highly conserved across species (Nikawa and Yamashita, 1992). As part of the MAM complex, the accumulation of IRE1α in MAM leads to either cell survival or cell death through the facilitation of excessive mitochondrial calcium levels (Shuda et al., 2003). Under normal conditions, the ER resident chaperone Bip is attached to the luminal domain of IRE1α, exerting negative regulation on it. During ER stress, Bip separates from IRE1, leading to the homodimerization and self-phosphorylation of IRE1. This is followed by the activation of its RNase domain. The activated IRE1 can then cleave the XBP1 precursor mRNA to generate the active spliced form of XBP1 (sXBP1). Once inside the nucleus, the sXBP1 mRNA is translated into the mature protein, which in turn stimulates the expression of genes related to protein folding and ultimately helps alleviate ER stress (Wang et al., 2022a). The likelihood of patients surviving IS is associated with the promotion of angiogenesis, and XBP1 plays a positive role in controlling the growth and development of brain microvascular endothelial cells following brain ischemia (Shi et al., 2019). A different research indicates that the IRE1/XBP1 pathway of the UPR becomes active during IS, and the lack of Xbp1 results in deteriorating stroke consequences (Jiang et al., 2017). Hence, IRE1 may modulate the occurrence of IS through the mediation of XBP1. Nevertheless, persistent and elevated levels of self-phosphorylation result in the formation of higher-order IRE1α oligomers, leading to a relaxation in the specificity of IRE1α. This subsequently triggers the degradation of numerous mRNAs encoding secretory proteins on the ER membrane through an intramolecular process known as regulated IRE1α-dependent decay (RIDD) (Oakes, 2017). RIDD plays a role in the degradation of various mRNAs and microRNAs, thereby regulating pathological processes such as inflammation and cell apoptosis (Wang et al., 2022a). Studies have demonstrated that continuous activation of IRE1α leads to neuronal death by reducing the expression of 14-3-3θ mRNA through IRE1α RIDD activity. Furthermore, IRE1α also serves as a scaffold to regulate the localization of inositol 1,4,5-trisphosphate receptors (IP3Rs) on MAM (Carreras-Sureda et al., 2019). Following an IS, the maintenance of intracellular calcium balance is essential for regulating neuronal function, with inositol 1,4,5-trisphosphate receptors (IP3Rs) serving as crucial channels for this purpose (Kesherwani and Agrawal, 2012). During cerebral ischemia, the ATP consumption is also linked to the continuous decrease in levels of inositol 1,4,5-trisphosphate, potentially resulting in neuronal function impairment and influencing cerebral ischemic injury (Sun and Hsu, 1996). IRE1α’s regulation of IP3Rs receptors has the potential to decrease neuronal mortality and subsequently control IS. In conclusion, the involvement of IRE1 in MAM is crucial for the regulation of ER stress, suggesting that targeting IRE1 in MAM could be a promising therapeutic strategy for IS (Table 6).

**Table 6:** The role of IRE1 in IS.

Intervention strategies	IRE1	ER stress	IS	References
Icariin	IRE1↓	Diminish	Prevent neuron apoptosis	Mo Z.T. et al. (2020)
Melatonin	IRE1↓	Diminish	Reducing acute neuronal damage after IS	Feng et al. (2017)
Longxue Tongluo capsule	IRE1↓	Diminish	Protecting brain neuron cells from ischemia–reperfusion injury	Pan et al. (2021)
Lumbrokinase	IRE1↓	Diminish	Improving Neurological Deficits in IS	Wang Y.H. et al. (2022)
Sodium formononetin-3′-sulphonate	IRE1↓	Diminish	The rate of cell apoptosis decreases.	Bai et al. (2022)
Enriched environment	IRE1↓	Diminish	Reduce acute neuronal damage	Han et al. (2023)

### 3.3 PERK in MAM and endoplasmic reticulum stress

The ER stress sensor PERK, a protein kinase R-like kinase, plays a crucial role in the UPR and is specifically abundant in MAM (Verfaillie et al., 2012). PERK plays a crucial role in MAM and has the ability to create a compound with S1R and Mfn2, contributing to the formation of MAM and enhancing the stability of ER-mitochondria interaction (Mao et al., 2022). One key sign of UPR activation following cerebral ischemia reperfusion involves the detection of PERK activation, which becomes fully active in the early stage of cerebral reperfusion (DeGracia and Montie, 2004). As a result of intracellular stress, unstructured or incorrectly folded proteins vie with GRP78 for binding, resulting in the dissociation of GRP78 from PERK. This leads to the release of inhibition on PERK and its activation through dimerization and self-phosphorylation (McQuiston and Diehl, 2017). Upon activation, PERK phosphorylates eukaryotic translation initiation factor 2 (eIF2α), leading to the inhibition of protein translation and a reduction in the aggregation of unfolded proteins within the ER lumen (Harding et al., 2000). PERK, a protein, is accountable for reducing mRNA translation during ER stress. This action helps to block the flow of newly produced proteins into the already stressed ER compartment. The process of translation attenuation is facilitated by the phosphorylation of eIF2α (Sano and Reed, 2013). During the early ischemia/reperfusion period, PERK significantly enhances the phosphorylation of eIF2α (Owen et al., 2005). In addition to its role in inhibiting protein translation, phosphorylated eIF2α is also capable of activating the expression of activated transcription factor 4 (ATF4) (Harding et al., 2000). ATF4 has the potential to influence cerebral ischemic injury through the regulation of cell apoptosis and neuronal function (Wu et al., 2022). Therefore, the PERK-ATF4 signaling pathway may play a role in neuronal apoptosis subsequent to IS. Apart from elF2α, PERK has the ability to phosphorylate nuclear red cell 2 p45-related factor 2 (NRF2) as well. The activation of NRF2 by PERK contributes to the maintenance of oxidation–reduction balance and promotes cell survival following ER stress (Cullinan and Diehl, 2004). During the occurrence of IS, NRF2 is involved in reducing oxidative stress, combating inflammation, regulating mitochondrial balance, and safeguarding the integrity of the blood–brain barrier to mitigate cerebral ischemic damage (Wang et al., 2022b). PERK may modulate IS through the phosphorylation of NRF2. Currently, there is a growing body of research investigating the role of PERK in the regulation of IS (refer to Table 7), and targeting PERK in MAM may hold promise as an approach for treating IS.

**Table 7:** The role of PERK in IS.

Intervention strategies	PERK	ER stress	IS	References
Melatonin	PERK↓	Diminish	Reducing acute neuronal damage after IS	Feng et al. (2017)
Ischemic preconditioning	PERK↓	Diminish	Reinforce the protection of the brain against ischemia–reperfusion injury.	Hu et al. (2017)
DL-3-n-butylphthalide	PERK↓	Diminish	Refraining from cerebral ischemia–reperfusion injury.	Zhu et al. (2018)
Dexmedetomidine	PERK↓	Diminish	Marked reduction in neuronal apoptosis.	Liu et al. (2018)
Scaffold protein Homer1a	PERK↓	Diminish	Reduce ER stress-induced mitochondrial stress after ischemia–reperfusion injury.	Wei et al. (2019)
Dantrolene	PERK↓	Diminish	It markedly decreases infarct volume and offers neuroprotection.	Li et al. (2005)
Astragaloside	PERK↓	Diminish	Reinforce the integrity of the blood–brain barrier and diminish the extent of the infarcted area.	Hou et al. (2020)

### 3.4 Sig-1R in MAM and endoplasmic reticulum stress

The sigma-1 receptor (Sig-1R) is a widely distributed co-factor found in the ER, specifically located in the MAM of the central nervous system. It plays a role in mediating signal transduction between the ER and mitochondria. Under normal conditions, Sig-1R is in a dormant state in MAM and forms a complex with another ER partner, BiP. During periods of ER stress, Sig-1R separates from BiP and controls the function of the three primary pathways of the UPR (PERK, IRE1a, ATF6) (Penke et al., 2018). The expression of Sig-1R is elevated in response to ER stress (Mitsuda et al., 2011). Overexpression of Sig-1R suppresses the activation of PERK and ATF6 signals, leading to enhanced cell survival (Hall et al., 2009). The decreased activity of Sig-1R destabilizes the conformation of IRE1a and diminishes cell viability (Mori et al., 2013). Agonists targeting Sig-1R have demonstrated protective effects on cells during stroke, and these effects are associated with the alleviation of ER stress (Morihara et al., 2018). It is evident that the upregulation of Sig-1R serves as a defensive mechanism for cell survival in response to ER stress. Moreover, Sig-1R has the capability to engage with inositol 1,4,5-trisphosphate receptor 3 (IP3R3) in order to stabilize its association with voltage-dependent anion channel 1 (VDAC1), thereby ensuring appropriate calcium transfer between the two cellular organelles (Morihara et al., 2018). After an IS, the downregulation of VDAC1 expression leads to intracellular calcium overload. Stabilizing the expression of VDAC1 can alleviate neuronal loss following cerebral ischemia (Yao et al., 2018). Sig-1R may stabilize VDAC1 through its interaction with IP3R3, thereby modulating the regulation of IS. It is important to mention that Sig-1R shows significant expression in the central nervous system, leading to its crucial involvement in functions such as cell differentiation, synapse growth, and the stimulation of microglia cells (Wu N.H. et al., 2021). The polarization of microglia/macrophages is a critical factor in the damage to tissues and the recovery of function following an IS. Additionally, Sig-1R serves as a crucial regulatory element in the macrophage-mediated clearance of deceased cells. The complete transplantation of Sig-1R macrophages has been shown to significantly decrease tissue damage and neurological impairments associated with IS (Zhang et al., 2023). In conclusion, targeting Sig-1R in MAM shows great promise for the treatment of IS (Table 8).

**Table 8:** The role of Sig-1R in IS.

Intervention strategies	Sig-1R	ER stress	IS	References
Dexmedetomidine	Sig-1R↑	Diminish	Reduce cerebral ischemia–reperfusion injury 24 h after brain injury	Zhai et al. (2019)
Sig-1R agonist	Sig-1R↑	Diminish	Promoting recovery after acute IS	Urfer et al. (2014)
Dimethyltryptamine	Sig-1R↑	Diminish	Inhibit cell apoptosis and enhance cell viability	Szabó et al. (2021)
Fluvoxamine	Sig-1R↑	Diminish	Refrain the expansion of infarcted area	Omi et al. (2014)

## 4 Discussion

IS has been one of the leading causes of death worldwide, and to date, there is no effective treatment to stop the progression of the disease. As the most direct interaction bridge between the ER and mitochondria, MAM plays a crucial role in regulating the activities carried out by these two organelles. In ubiquitin-dependent mitochondrial autophagy, PINK1/Parkin is enriched in MAM. After IS, PINK1/Parkin pathway is activated to induce mitochondrial autophagy through Mfn2 ubiquitination. Beclin1 promotes mitochondrial autophagy by promoting the translocation of Parkin. As one of the non-ubiquitin-dependent mitochondrial autophagy, FUNDC1 is enriched in MAM, and phosphorylation of FUNDC1 under hypoxia affects its binding to LC3, thereby mediating mitochondrial autophagy. ER stress induced by IS can enhance the transcriptional rate of GRP78, dissociation of GRP78 from UPR leads to activation of PERK and IRE1, and upregulation of Sig-1R, thus affecting ER function and cell survival. In addition, more and more studies have shown that MAM plays an important role in regulating inflammation, oxidative stress, lipid metabolism, membrane dynamics, calcium signaling, etc. Thus, as an interface between energy metabolism, protein homeostasis, and cell fate control, MAM-mediated mitochondrial autophagy and ER stress play an important role in cell survival in the brain. MAM may be an important target for regulating IS and has important significance for improving the survival rate of patients.

However, despite the summary of many relevant molecules involved in the regulation of IS in the MAM, many questions remain to be addressed. The role of MAM in IS has not received enough attention, and the signaling pathway of MAM regulating IS has not been studied. Metabolic syndrome (MetS) is a series of interrelated vascular risk factors (including insulin resistance/diabetes, hypertension, central obesity, dyslipidemia) that are positively associated with adverse outcomes of IS and may lead to cerebrovascular damage and deterioration of neurofunction (Zhang et al., 2016). It is worth noting that numerous studies have found that MAM is involved in the regulation of metabolic syndrome: 1. mTOR, the mammalian target of rapamycin, is involved in a series of physiological and pathological processes of insulin signal transduction, and mTOR is located in MAM (Xu et al., 2017). Disruption of MAM integrity affects insulin resistance (Tubbs et al., 2018). 2. Gopinath et al. confirmed direct evidence that MAM is involved in the development of hypertension, and the Nogo-B family of reticulum proteins after pulmonary hypertension destroys MAM and inhibits apoptosis (Sutendra et al., 2011). 3. Enzymes involved in lipid metabolism are the most abundant proteins on MAM (Vance, 2014). 4. MAM plays a potential role in fat storage by transporting sufficient lipids to mitochondria for β-oxidation during fasting (Theurey et al., 2016). 5. PINK1/Parkin and FUNDC1 regulate the development of MetS by exerting anti-inflammatory and antioxidant stress effects through mitochondrial autophagy (Miao et al., 2023). But the link between MAM and IS and MetS has not been thoroughly studied. In the future, it may be a new research direction to explore the mechanism that can improve metabolic function, reduce ischemic injury and promote the recovery of neurological function.
